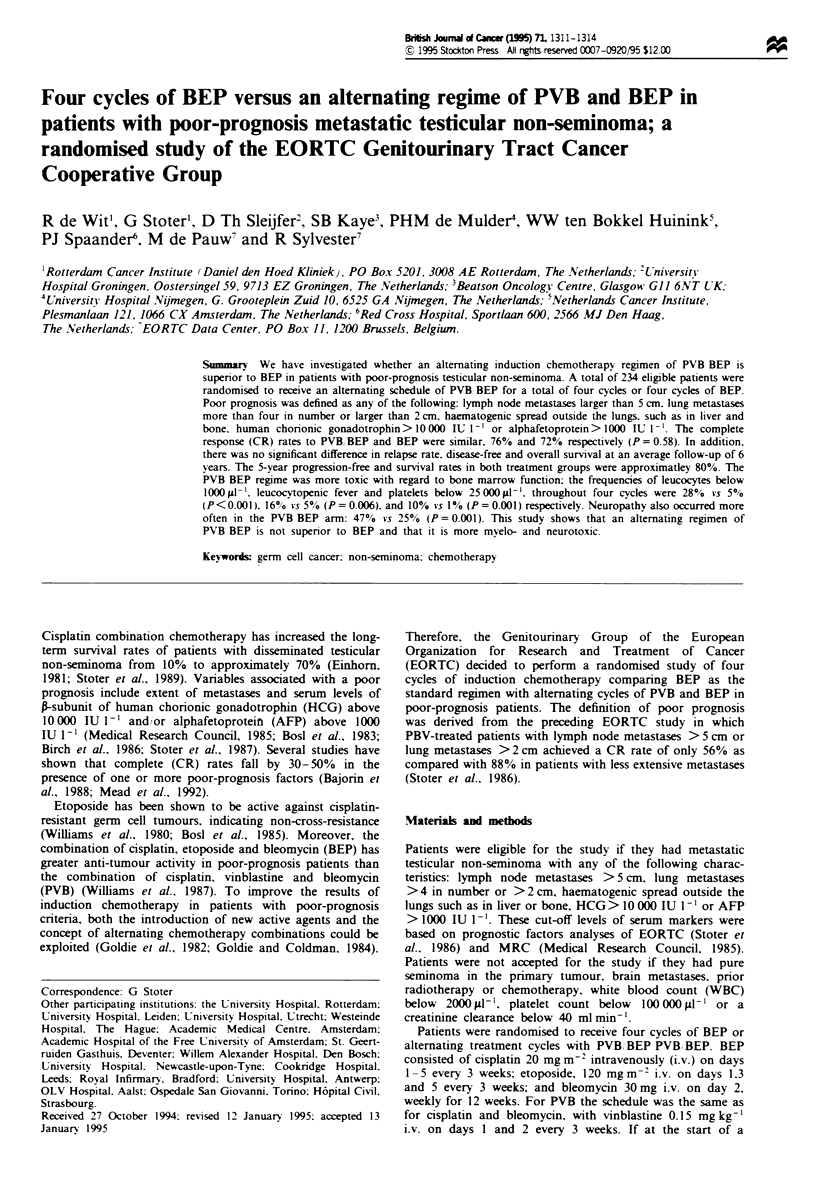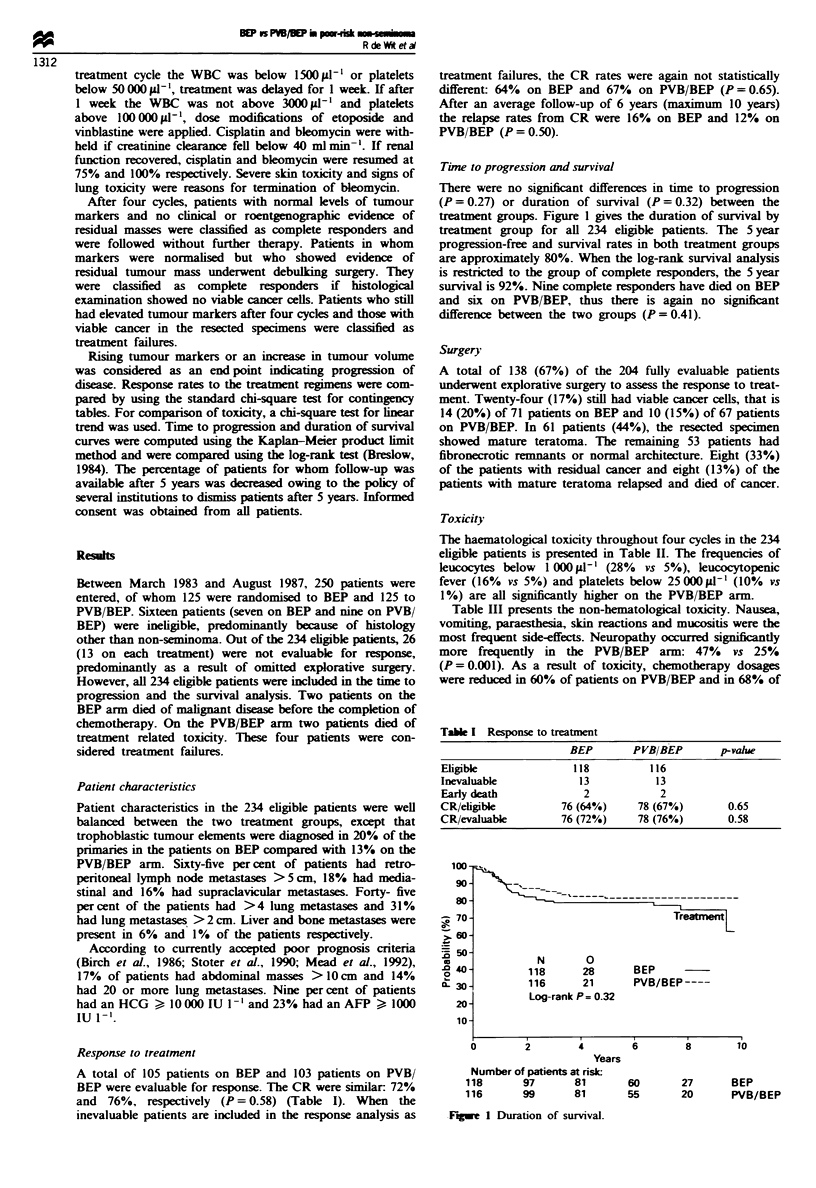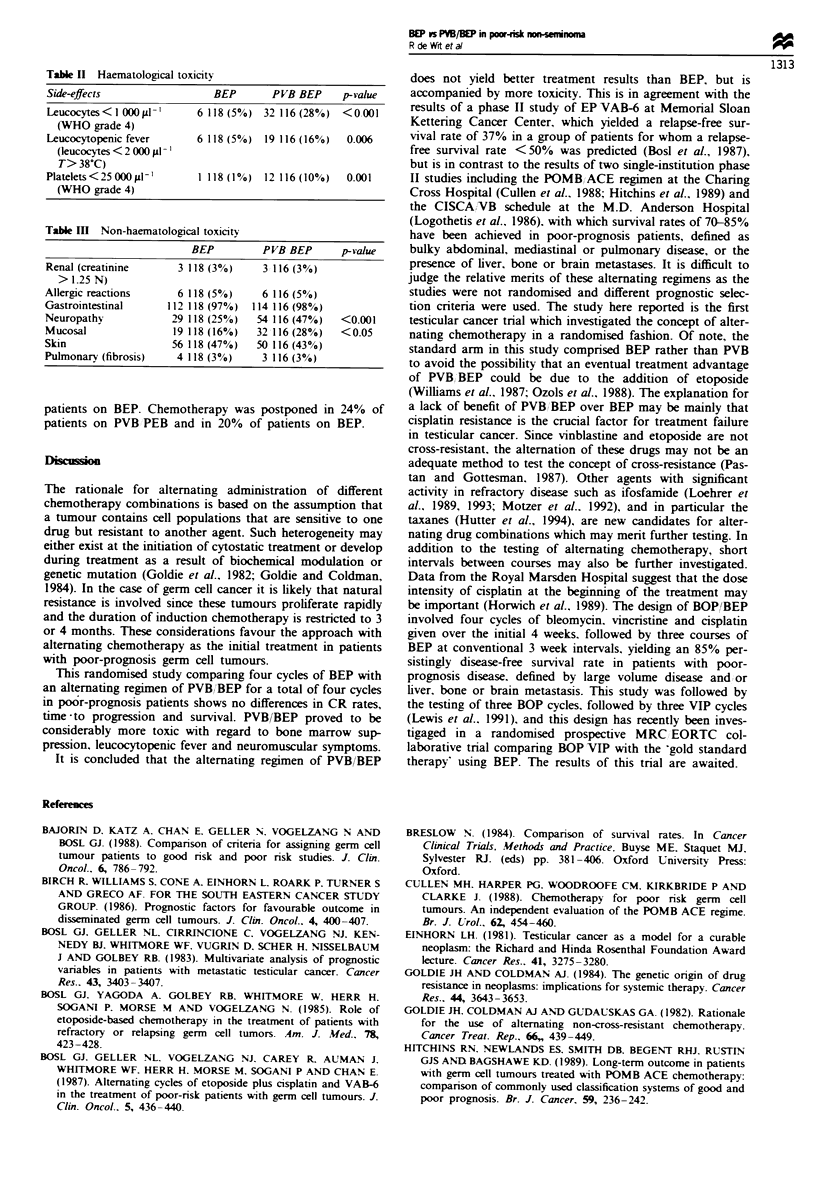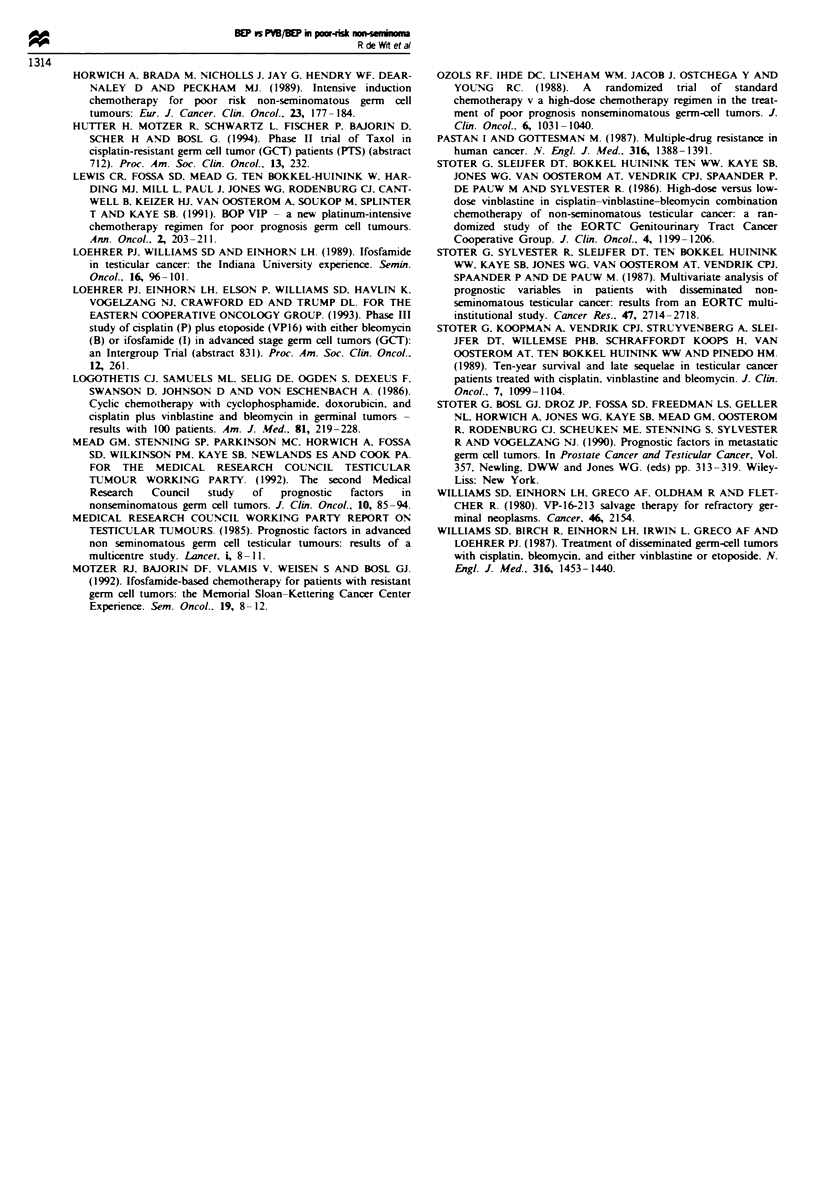# Four cycles of BEP versus an alternating regime of PVB and BEP in patients with poor-prognosis metastatic testicular non-seminoma; a randomised study of the EORTC Genitourinary Tract Cancer Cooperative Group.

**DOI:** 10.1038/bjc.1995.254

**Published:** 1995-06

**Authors:** R. de Wit, G. Stoter, D. T. Sleijfer, S. B. Kaye, P. H. de Mulder, W. W. ten Bokkel Huinink, P. J. Spaander, M. de Pauw, R. Sylvester

**Affiliations:** Rotterdam Cancer Institute (Daniel den Hoed Kliniek), The Netherlands.

## Abstract

We have investigated whether an alternating induction chemotherapy regimen of PVB/BEP is superior to BEP in patients with poor-prognosis testicular non-seminoma. A total of 234 eligible patients were randomised to receive an alternating schedule of PVB/BEP for a total of four cycles or four cycles of BEP. Poor prognosis was defined as any of the following: lymph node metastases larger than 5 cm, lung metastases more than four in number or larger than 2 cm, haematogenic spread outside the lungs, such as in liver and bone, human chorionic gonadotrophin > 10,000 IU l-1 or alphafetoprotein > 1000 IU l-1. The complete response (CR) rates to PVB/BEP and BEP were similar, 76% and 72% respectively (P = 0.58). In addition, there was no significant difference in relapse rate, disease-free and overall survival at an average follow-up of 6 years. The 5-year progression-free and survival rates in both treatment groups were approximately 80%. The PVB/BEP regime was more toxic with regard to bone marrow function; the frequencies of leucocytes below 1000 microliters-1, leucocytopenic fever and platelets below 25,000 microliters-1, throughout four cycles were 28% vs 5% (P < 0.001), 16% vs 5% (P = 0.006), and 10% vs 1% (P = 0.001) respectively. Neuropathy also occurred more often in the PVB/BEP arm: 47% vs 25% (P = 0.001). This study shows that an alternating regimen of PVB/BEP is not superior to BEP and that it is more myelo- and neurotoxic.


© 1995 Stockton Press All rights reserved 0007-0920/95 $12.00

### Four cycles of BEP versus an alternating regime of PVB and BEP in patients with poor-prognosis metastatic testicular non-seminoma; a randomised study of the EORTC Genitourinary Tract Cancer Cooperative Group

R de Wit[1], G Stoter[1], D Th Sleijfer[2], SB Kaye[3], PHM de Mulder[4], WW ten Bokkel Huinink[5], PJ Spaander[6], M de Pauw[7] and R Sylvester[7]

[1]*Rotterdam Cancer Institute (Daniel den Hoed Kliniek), PO Box 5201, 3008 AE Rotterdam, The Netherlands;* [2]*University Hospital Groningen, Oostersingel 59, 9713 EZ Groningen, The Netherlands;* [3]*Beatson Oncology Centre, Glasgow G11 6NT UK;* [4]*University Hospital Nijmegen, G. Grooteplein Zuid 10, 6525 GA Nijmegen, The Netherlands;* [5]*Netherlands Cancer Institute, Plesmanlaan 121, 1066 CX Amsterdam, The Netherlands;* [6]*Red Cross Hospital, Sportlaan 600, 2566 MJ Den Haag, The Netherlands;* [7]*EORTC Data Center, PO Box 11, 1200 Brussels, Belgium.*

**Summary** We have investigated whether an alternating induction chemotherapy regimen of PVB/BEP is superior to BEP in patients with poor-prognosis testicular non-seminoma. A total of 234 eligible patients were randomised to receive an alternating schedule of PVB/BEP for a total of four cycles or four cycles of BEP. Poor prognosis was defined as any of the following: lymph node metastases larger than 5 cm, lung metastases more than four in number or larger than 2 cm, haematogenic spread outside the lungs, such as in liver and bone, human chorionic gonadotrophin $>10\,000$ IU $l^{-1}$ or alphafetoprotein $>1000$ IU $l^{-1}$. The complete response (CR) rates to PVB/BEP and BEP were similar, 76% and 72% respectively ($P=0.58$). In addition, there was no significant difference in relapse rate, disease-free and overall survival at an average follow-up of 6 years. The 5-year progression-free and survival rates in both treatment groups were approximatley 80%. The PVB/BEP regime was more toxic with regard to bone marrow function; the frequencies of leucocytes below 1000 $\mu l^{-1}$, leucocytopenic fever and platelets below 25 000 $\mu l^{-1}$, throughout four cycles were 28% *vs* 5% ($P<0.001$), 16% *vs* 5% ($P=0.006$), and 10% *vs* 1% ($P=0.001$) respectively. Neuropathy also occurred more often in the PVB/BEP arm: 47% *vs* 25% ($P=0.001$). This study shows that an alternating regimen of PVB/BEP is not superior to BEP and that it is more myelo- and neurotoxic.

**Keywords:** germ cell cancer; non-seminoma; chemotherapy

Cisplatin combination chemotherapy has increased the long-term survival rates of patients with disseminated testicular non-seminoma from 10% to approximately 70% (Einhorn, 1981; Stoter *et al.*, 1989). Variables associated with a poor prognosis include extent of metastases and serum levels of β-subunit of human chorionic gonadotrophin (HCG) above 10 000 IU $l^{-1}$ and/or alphafetoprotein (AFP) above 1000 IU $l^{-1}$ (Medical Research Council, 1985; Bosl *et al.*, 1983; Birch *et al.*, 1986; Stoter *et al.*, 1987). Several studies have shown that complete (CR) rates fall by 30-50% in the presence of one or more poor-prognosis factors (Bajorin *et al.*, 1988; Mead *et al.*, 1992).

Etoposide has been shown to be active against cisplatin-resistant germ cell tumours, indicating non-cross-resistance (Williams *et al.*, 1980; Bosl *et al.*, 1985). Moreover, the combination of cisplatin, etoposide and bleomycin (BEP) has greater anti-tumour activity in poor-prognosis patients than the combination of cisplatin, vinblastine and bleomycin (PVB) (Williams *et al.*, 1987). To improve the results of induction chemotherapy in patients with poor-prognosis criteria, both the introduction of new active agents and the concept of alternating chemotherapy combinations could be exploited (Goldie *et al.*, 1982; Goldie and Coldman, 1984).

Therefore, the Genitourinary Group of the European Organization for Research and Treatment of Cancer (EORTC) decided to perform a randomised study of four cycles of induction chemotherapy comparing BEP as the standard regimen with alternating cycles of PVB and BEP in poor-prognosis patients. The definition of poor prognosis was derived from the preceding EORTC study in which PBV-treated patients with lymph node metastases $>5$ cm or lung metastases $>2$ cm achieved a CR rate of only 56% as compared with 88% in patients with less extensive metastases (Stoter *et al.*, 1986).

### Materials and methods

Patients were eligible for the study if they had metastatic testicular non-seminoma with any of the following characteristics: lymph node metastases $>5$ cm, lung metastases $>4$ in number or $>2$ cm, haematogenic spread outside the lungs such as in liver or bone, HCG $>10\,000$ IU $l^{-1}$ or AFP $>1000$ IU $l^{-1}$. These cut-off levels of serum markers were based on prognostic factors analyses of EORTC (Stoter *et al.*, 1986) and MRC (Medical Research Council, 1985). Patients were not accepted for the study if they had pure seminoma in the primary tumour, brain metastases, prior radiotherapy or chemotherapy, white blood count (WBC) below 2000 $\mu l^{-1}$, platelet count below 100 000 $\mu l^{-1}$ or a creatinine clearance below 40 ml $min^{-1}$.

Patients were randomised to receive four cycles of BEP or alternating treatment cycles with PVB/BEP/PVB/BEP. BEP consisted of cisplatin 20 mg $m^{-2}$ intravenously (i.v.) on days 1-5 every 3 weeks; etoposide, 120 mg $m^{-2}$ i.v. on days 1,3 and 5 every 3 weeks; and bleomycin 30 mg i.v. on day 2, weekly for 12 weeks. For PVB the schedule was the same as for cisplatin and bleomycin, with vinblastine 0.15 mg $kg^{-1}$ i.v. on days 1 and 2 every 3 weeks. If at the start of a

Correspondence: G Stoter
Other participating institutions: the University Hospital, Rotterdam; University Hospital, Leiden; University Hospital, Utrecht; Westeinde Hospital, The Hague; Academic Medical Centre, Amsterdam; Academic Hospital of the Free University of Amsterdam; St. Geertruiden Gasthuis, Deventer; Willem Alexander Hospital, Den Bosch; University Hospital, Newcastle-upon-Tyne; Cookridge Hospital, Leeds; Royal Infirmary, Bradford; University Hospital, Antwerp; OLV Hospital, Aalst; Ospedale San Giovanni, Torino; Hôpital Civil, Strasbourg.
Received 27 October 1994; revised 12 January 1995; accepted 13 January 1995

treatment cycle the WBC was below 1500 $\mu l^{-1}$ or platelets below 50 000 $\mu l^{-1}$, treatment was delayed for 1 week. If after 1 week the WBC was not above 3000 $\mu l^{-1}$ and platelets above 100 000 $\mu l^{-1}$, dose modifications of etoposide and vinblastine were applied. Cisplatin and bleomycin were withheld if creatinine clearance fell below 40 ml $min^{-1}$. If renal function recovered, cisplatin and bleomycin were resumed at 75% and 100% respectively. Severe skin toxicity and signs of lung toxicity were reasons for termination of bleomycin.

After four cycles, patients with normal levels of tumour markers and no clinical or roentgenographic evidence of residual masses were classified as complete responders and were followed without further therapy. Patients in whom markers were normalised but who showed evidence of residual tumour mass underwent debulking surgery. They were classified as complete responders if histological examination showed no viable cancer cells. Patients who still had elevated tumour markers after four cycles and those with viable cancer in the resected specimens were classified as treatment failures.

Rising tumour markers or an increase in tumour volume was considered as an end point indicating progression of disease. Response rates to the treatment regimens were compared by using the standard chi-square test for contingency tables. For comparison of toxicity, a chi-square test for linear trend was used. Time to progression and duration of survival curves were computed using the Kaplan–Meier product limit method and were compared using the log-rank test (Breslow, 1984). The percentage of patients for whom follow-up was available after 5 years was decreased owing to the policy of several institutions to dismiss patients after 5 years. Informed consent was obtained from all patients.

### Results

Between March 1983 and August 1987, 250 patients were entered, of whom 125 were randomised to BEP and 125 to PVB/BEP. Sixteen patients (seven on BEP and nine on PVB/BEP) were ineligible, predominantly because of histology other than non-seminoma. Out of the 234 eligible patients, 26 (13 on each treatment) were not evaluable for response, predominantly as a result of omitted explorative surgery. However, all 234 eligible patients were included in the time to progression and the survival analysis. Two patients on the BEP arm died of malignant disease before the completion of chemotherapy. On the PVB/BEP arm two patients died of treatment related toxicity. These four patients were considered treatment failures.

### *Patient characteristics*

Patient characteristics in the 234 eligible patients were well balanced between the two treatment groups, except that trophoblastic tumour elements were diagnosed in 20% of the primaries in the patients on BEP compared with 13% on the PVB/BEP arm. Sixty-five per cent of patients had retroperitoneal lymph node metastases >5 cm, 18% had mediastinal and 16% had supraclavicular metastases. Forty- five per cent of the patients had >4 lung metastases and 31% had lung metastases >2 cm. Liver and bone metastases were present in 6% and 1% of the patients respectively.

According to currently accepted poor prognosis criteria (Birch *et al.*, 1986; Stoter *et al.*, 1990; Mead *et al.*, 1992), 17% of patients had abdominal masses >10 cm and 14% had 20 or more lung metastases. Nine per cent of patients had an HCG ⩾ 10 000 IU $l^{-1}$ and 23% had an AFP ⩾ 1000 IU $l^{-1}$.

### *Response to treatment*

A total of 105 patients on BEP and 103 patients on PVB/BEP were evaluable for response. The CR were similar: 72% and 76%, respectively ($P = 0.58$) (Table I). When the inevaluable patients are included in the response analysis as treatment failures, the CR rates were again not statistically different: 64% on BEP and 67% on PVB/BEP ($P = 0.65$). After an average follow-up of 6 years (maximum 10 years) the relapse rates from CR were 16% on BEP and 12% on PVB/BEP ($P = 0.50$).

### *Time to progression and survival*

There were no significant differences in time to progression ($P = 0.27$) or duration of survival ($P = 0.32$) between the treatment groups. Figure 1 gives the duration of survival by treatment group for all 234 eligible patients. The 5 year progression-free and survival rates in both treatment groups are approximately 80%. When the log-rank survival analysis is restricted to the group of complete responders, the 5 year survival is 92%. Nine complete responders have died on BEP and six on PVB/BEP, thus there is again no significant difference between the two groups ($P = 0.41$).

### *Surgery*

A total of 138 (67%) of the 204 fully evaluable patients underwent explorative surgery to assess the response to treatment. Twenty-four (17%) still had viable cancer cells, that is 14 (20%) of 71 patients on BEP and 10 (15%) of 67 patients on PVB/BEP. In 61 patients (44%), the resected specimen showed mature teratoma. The remaining 53 patients had fibronecrotic remnants or normal architecture. Eight (33%) of the patients with residual cancer and eight (13%) of the patients with mature teratoma relapsed and died of cancer.

### *Toxicity*

The haematological toxicity throughout four cycles in the 234 eligible patients is presented in Table II. The frequencies of leucocytes below 1 000 $\mu l^{-1}$ (28% *vs* 5%), leucocytopenic fever (16% *vs* 5%) and platelets below 25 000 $\mu l^{-1}$ (10% *vs* 1%) are all significantly higher on the PVB/BEP arm.

Table III presents the non-hematological toxicity. Nausea, vomiting, paraesthesia, skin reactions and mucositis were the most frequent side-effects. Neuropathy occurred significantly more frequently in the PVB/BEP arm: 47% *vs* 25% ($P = 0.001$). As a result of toxicity, chemotherapy dosages were reduced in 60% of patients on PVB/BEP and in 68% of

**Table I** Response to treatment

| | *BEP* | *PVB/BEP* | *p-value* |
|---|---|---|---|
| Eligible | 118 | 116 | |
| Inevaluable | 13 | 13 | |
| Early death | 2 | 2 | |
| CR/eligible | 76 (64%) | 78 (67%) | 0.65 |
| CR/evaluable | 76 (72%) | 78 (76%) | 0.58 |

**Figure 1** Duration of survival.

**Table II** Haematological toxicity

| *Side-effects* | *BEP* | *PVB BEP* | *p-value* |
|---|---|---|---|
| Leucocytes $< 1\,000\ \mu l^{-1}$ (WHO grade 4) | 6 118 (5%) | 32 116 (28%) | <0.001 |
| Leucocytopenic fever (leucocytes $< 2\,000\ \mu l^{-1}$ $T > 38°C$) | 6 118 (5%) | 19 116 (16%) | 0.006 |
| Platelets $< 25\,000\ \mu l^{-1}$ (WHO grade 4) | 1 118 (1%) | 12 116 (10%) | 0.001 |

**Table III** Non-haematological toxicity

| | *BEP* | *PVB BEP* | *p-value* |
|---|---|---|---|
| Renal (creatinine > 1.25 N) | 3 118 (3%) | 3 116 (3%) | |
| Allergic reactions | 6 118 (5%) | 6 116 (5%) | |
| Gastrointestinal | 112 118 (97%) | 114 116 (98%) | |
| Neuropathy | 29 118 (25%) | 54 116 (47%) | <0.001 |
| Mucosal | 19 118 (16%) | 32 116 (28%) | <0.05 |
| Skin | 56 118 (47%) | 50 116 (43%) | |
| Pulmonary (fibrosis) | 4 118 (3%) | 3 116 (3%) | |

patients on BEP. Chemotherapy was postponed in 24% of patients on PVB PEB and in 20% of patients on BEP.

### Discussion

The rationale for alternating administration of different chemotherapy combinations is based on the assumption that a tumour contains cell populations that are sensitive to one drug but resistant to another agent. Such heterogeneity may either exist at the initiation of cytostatic treatment or develop during treatment as a result of biochemical modulation or genetic mutation (Goldie *et al.*, 1982; Goldie and Coldman, 1984). In the case of germ cell cancer it is likely that natural resistance is involved since these tumours proliferate rapidly and the duration of induction chemotherapy is restricted to 3 or 4 months. These considerations favour the approach with alternating chemotherapy as the initial treatment in patients with poor-prognosis germ cell tumours.

This randomised study comparing four cycles of BEP with an alternating regimen of PVB/BEP for a total of four cycles in poor-prognosis patients shows no differences in CR rates, time to progression and survival. PVB/BEP proved to be considerably more toxic with regard to bone marrow suppression, leucocytopenic fever and neuromuscular symptoms.

It is concluded that the alternating regimen of PVB/BEP does not yield better treatment results than BEP, but is accompanied by more toxicity. This is in agreement with the results of a phase II study of EP/VAB-6 at Memorial Sloan Kettering Cancer Center, which yielded a relapse-free survival rate of 37% in a group of patients for whom a relapse-free survival rate <50% was predicted (Bosl *et al.*, 1987), but is in contrast to the results of two single-institution phase II studies including the POMB/ACE regimen at the Charing Cross Hospital (Cullen *et al.*, 1988; Hitchins *et al.*, 1989) and the CISCA/VB schedule at the M.D. Anderson Hospital (Logothetis *et al.*, 1986), with which survival rates of 70–85% have been achieved in poor-prognosis patients, defined as bulky abdominal, mediastinal or pulmonary disease, or the presence of liver, bone or brain metastases. It is difficult to judge the relative merits of these alternating regimens as the studies were not randomised and different prognostic selection criteria were used. The study here reported is the first testicular cancer trial which investigated the concept of alternating chemotherapy in a randomised fashion. Of note, the standard arm in this study comprised BEP rather than PVB to avoid the possibility that an eventual treatment advantage of PVB/BEP could be due to the addition of etoposide (Williams *et al.*, 1987; Ozols *et al.*, 1988). The explanation for a lack of benefit of PVB/BEP over BEP may be mainly that cisplatin resistance is the crucial factor for treatment failure in testicular cancer. Since vinblastine and etoposide are not cross-resistant, the alternation of these drugs may not be an adequate method to test the concept of cross-resistance (Pastan and Gottesman, 1987). Other agents with significant activity in refractory disease such as ifosfamide (Loehrer *et al.*, 1989, 1993; Motzer *et al.*, 1992), and in particular the taxanes (Hutter *et al.*, 1994), are new candidates for alternating drug combinations which may merit further testing. In addition to the testing of alternating chemotherapy, short intervals between courses may also be further investigated. Data from the Royal Marsden Hospital suggest that the dose intensity of cisplatin at the beginning of the treatment may be important (Horwich *et al.*, 1989). The design of BOP/BEP involved four cycles of bleomycin, vincristine and cisplatin given over the initial 4 weeks, followed by three courses of BEP at conventional 3 week intervals, yielding an 85% persistingly disease-free survival rate in patients with poor-prognosis disease, defined by large volume disease and/or liver, bone or brain metastasis. This study was followed by the testing of three BOP cycles, followed by three VIP cycles (Lewis *et al.*, 1991), and this design has recently been investigaged in a randomised prospective MRC/EORTC collaborative trial comparing BOP/VIP with the 'gold standard therapy' using BEP. The results of this trial are awaited.

### References

BAJORIN D, KATZ A, CHAN E, GELLER N, VOGELZANG N AND BOSL GJ. (1988). Comparison of criteria for assigning germ cell tumour patients to good risk and poor risk studies. *J. Clin. Oncol.*, **6**, 786–792.

BIRCH R, WILLIAMS S, CONE A, EINHORN L, ROARK P, TURNER S AND GRECO AF. FOR THE SOUTH EASTERN CANCER STUDY GROUP. (1986). Prognostic factors for favourable outcome in disseminated germ cell tumours. *J. Clin. Oncol.*, **4**, 400–407.

BOSL GJ, GELLER NL, CIRRINCIONE C, VOGELZANG NJ, KENNEDY BJ, WHITMORE WF, VUGRIN D, SCHER H, NISSELBAUM J AND GOLBEY RB. (1983). Multivariate analysis of prognostic variables in patients with metastatic testicular cancer. *Cancer Res.*, **43**, 3403–3407.

BOSL GJ, YAGODA A, GOLBEY RB, WHITMORE W, HERR H, SOGANI P, MORSE M AND VOGELZANG N. (1985). Role of etoposide-based chemotherapy in the treatment of patients with refractory or relapsing germ cell tumors. *Am. J. Med.*, **78**, 423–428.

BOSL GJ, GELLER NL, VOGELZANG NJ, CAREY R, AUMAN J, WHITMORE WF, HERR H, MORSE M, SOGANI P AND CHAN E. (1987). Alternating cycles of etoposide plus cisplatin and VAB-6 in the treatment of poor-risk patients with germ cell tumours. *J. Clin. Oncol.*, **5**, 436–440.

BRESLOW N. (1984). Comparison of survival rates. In *Cancer Clinical Trials, Methods and Practice*, Buyse ME, Staquet MJ, Sylvester RJ. (eds) pp. 381–406. Oxford University Press: Oxford.

CULLEN MH, HARPER PG, WOODROOFE CM, KIRKBRIDE P AND CLARKE J. (1988). Chemotherapy for poor risk germ cell tumours. An independent evaluation of the POMB ACE regime. *Br. J. Urol.*, **62**, 454–460.

EINHORN LH. (1981). Testicular cancer as a model for a curable neoplasm: the Richard and Hinda Rosenthal Foundation Award lecture. *Cancer Res.*, **41**, 3275–3280.

GOLDIE JH AND COLDMAN AJ. (1984). The genetic origin of drug resistance in neoplasms: implications for systemic therapy. *Cancer Res.*, **44**, 3643–3653.

GOLDIE JH, COLDMAN AJ AND GUDAUSKAS GA. (1982). Rationale for the use of alternating non-cross-resistant chemotherapy. *Cancer Treat. Rep.*, **66**, 439–449.

HITCHINS RN, NEWLANDS ES, SMITH DB, BEGENT RHJ, RUSTIN GJS AND BAGSHAWE KD. (1989). Long-term outcome in patients with germ cell tumours treated with POMB ACE chemotherapy: comparison of commonly used classification systems of good and poor prognosis. *Br. J. Cancer*, **59**, 236–242.

HORWICH A, BRADA M, NICHOLLS J, JAY G, HENDRY WF, DEARNALEY D AND PECKHAM MJ. (1989). Intensive induction chemotherapy for poor risk non-seminomatous germ cell tumours: *Eur. J. Cancer. Clin. Oncol.*, **23**, 177-184.

HUTTER H, MOTZER R, SCHWARTZ L, FISCHER P, BAJORIN D, SCHER H AND BOSL G. (1994). Phase II trial of Taxol in cisplatin-resistant germ cell tumor (GCT) patients (PTS) (abstract 712). *Proc. Am. Soc. Clin. Oncol.*, **13**, 232.

LEWIS CR, FOSSA SD, MEAD G, TEN BOKKEL-HUININK W, HARDING MJ, MILL L, PAUL J, JONES WG, RODENBURG CJ, CANTWELL B, KEIZER HJ, VAN OOSTEROM A, SOUKOP M, SPLINTER T AND KAYE SB. (1991). BOP/VIP - a new platinum-intensive chemotherapy regimen for poor prognosis germ cell tumours. *Ann. Oncol.*, **2**, 203-211.

LOEHRER PJ, WILLIAMS SD AND EINHORN LH. (1989). Ifosfamide in testicular cancer: the Indiana University experience. *Semin. Oncol.*, **16**, 96-101.

LOEHRER PJ, EINHORN LH, ELSON P, WILLIAMS SD, HAVLIN K, VOGELZANG NJ, CRAWFORD ED AND TRUMP DL. FOR THE EASTERN COOPERATIVE ONCOLOGY GROUP. (1993). Phase III study of cisplatin (P) plus etoposide (VP16) with either bleomycin (B) or ifosfamide (I) in advanced stage germ cell tumors (GCT): an Intergroup Trial (abstract 831). *Proc. Am. Soc. Clin. Oncol.*, **12**, 261.

LOGOTHETIS CJ, SAMUELS ML, SELIG DE, OGDEN S, DEXEUS F, SWANSON D, JOHNSON D AND VON ESCHENBACH A. (1986). Cyclic chemotherapy with cyclophosphamide, doxorubicin, and cisplatin plus vinblastine and bleomycin in germinal tumors - results with 100 patients. *Am. J. Med.*, **81**, 219-228.

MEAD GM, STENNING SP, PARKINSON MC, HORWICH A, FOSSA SD, WILKINSON PM, KAYE SB, NEWLANDS ES AND COOK PA. FOR THE MEDICAL RESEARCH COUNCIL TESTICULAR TUMOUR WORKING PARTY. (1992). The second Medical Research Council study of prognostic factors in nonseminomatous germ cell tumors. *J. Clin. Oncol.*, **10**, 85-94.

MEDICAL RESEARCH COUNCIL WORKING PARTY REPORT ON TESTICULAR TUMOURS. (1985). Prognostic factors in advanced non seminomatous germ cell testicular tumours: results of a multicentre study. *Lancet*, **i**, 8-11.

MOTZER RJ, BAJORIN DF, VLAMIS V, WEISEN S AND BOSL GJ. (1992). Ifosfamide-based chemotherapy for patients with resistant germ cell tumors: the Memorial Sloan-Kettering Cancer Center Experience. *Sem. Oncol.*, **19**, 8-12.

OZOLS RF, IHDE DC, LINEHAM WM, JACOB J, OSTCHEGA Y AND YOUNG RC. (1988). A randomized trial of standard chemotherapy v a high-dose chemotherapy regimen in the treatment of poor prognosis nonseminomatous germ-cell tumors. *J. Clin. Oncol.*, **6**, 1031-1040.

PASTAN I AND GOTTESMAN M. (1987). Multiple-drug resistance in human cancer. *N. Engl. J. Med.*, **316**, 1388-1391.

STOTER G, SLEIJFER DT, BOKKEL HUININK TEN WW, KAYE SB, JONES WG, VAN OOSTEROM AT, VENDRIK CPJ, SPAANDER P, DE PAUW M AND SYLVESTER R. (1986). High-dose versus low-dose vinblastine in cisplatin-vinblastine-bleomycin combination chemotherapy of non-seminomatous testicular cancer: a randomized study of the EORTC Genitourinary Tract Cancer Cooperative Group. *J. Clin. Oncol.*, **4**, 1199-1206.

STOTER G, SYLVESTER R, SLEIJFER DT, TEN BOKKEL HUININK WW, KAYE SB, JONES WG, VAN OOSTEROM AT, VENDRIK CPJ, SPAANDER P AND DE PAUW M. (1987). Multivariate analysis of prognostic variables in patients with disseminated non-seminomatous testicular cancer: results from an EORTC multi-institutional study. *Cancer Res.*, **47**, 2714-2718.

STOTER G, KOOPMAN A, VENDRIK CPJ, STRUYVENBERG A, SLEIJFER DT, WILLEMSE PHB, SCHRAFFORDT KOOPS H, VAN OOSTEROM AT, TEN BOKKEL HUININK WW AND PINEDO HM. (1989). Ten-year survival and late sequelae in testicular cancer patients treated with cisplatin, vinblastine and bleomycin. *J. Clin. Oncol.*, **7**, 1099-1104.

STOTER G, BOSL GJ, DROZ JP, FOSSA SD, FREEDMAN LS, GELLER NL, HORWICH A, JONES WG, KAYE SB, MEAD GM, OOSTEROM R, RODENBURG CJ, SCHEUKEN ME, STENNING S, SYLVESTER R AND VOGELZANG NJ. (1990). Prognostic factors in metastatic germ cell tumors. In *Prostate Cancer and Testicular Cancer*, Vol. 357, Newling, DWW and Jones WG. (eds) pp. 313-319. Wiley-Liss: New York.

WILLIAMS SD, EINHORN LH, GRECO AF, OLDHAM R AND FLETCHER R. (1980). VP-16-213 salvage therapy for refractory germinal neoplasms. *Cancer*, **46**, 2154.

WILLIAMS SD, BIRCH R, EINHORN LH, IRWIN L, GRECO AF AND LOEHRER PJ. (1987). Treatment of disseminated germ-cell tumors with cisplatin, bleomycin, and either vinblastine or etoposide. *N. Engl. J. Med.*, **316**, 1453-1440.